# Preparing for tenure and promotion at PUI institutions

**DOI:** 10.1186/s12919-021-00219-2

**Published:** 2021-06-22

**Authors:** Leticia R. Vega, Christoph J. Hengartner

**Affiliations:** Department of Biology, Barry University, Miami Shores, FL USA

**Keywords:** Promotion, Tenure, PUI, Faculty, Mentoring, Evaluation, Teaching, Dossier, Documentation, Planning, Service, Research

## Abstract

In this paper, we discuss the importance for faculty to become familiar with the general guidelines for collecting, assembling and preparing a tenure and promotion (T&P) application or dossier at a Primarily Undergraduate Institution (PUI) and the critical role that mentoring plays throughout the T&P process. While key elements of the application process such as submission timelines and documentation guidelines are usually outlined in the faculty handbook of the specific institution, many aspects of assembling the dossier are not necessarily detailed in writing anywhere. Instead, there are important elements of the T&P process that typically rely on institutional knowledge and guidance that is often communicated informally. Junior faculty who have limited access to “informal communications” are at a significant disadvantage when they go through the T&P process even when they show accomplishments in teaching effectiveness, research, and service. The problem is especially important for women and underrepresented minority faculty in STEM disciplines that are less well represented among senior faculty in STEM. Senior faculty often serve as informal or formal mentors to their less seasoned colleagues. The goal of this article is to help demystify the T&P process by offering practical suggestions and describing some of the specific materials and steps that are an important part of documenting the development of a faculty member at a PUI.

## Background

This paper will focus on key aspects of the T&P process at Primarily Undergraduate Institutions (PUI) designated by the Carnegie Classifications system [1], including liberal arts colleges. An article by Boyce and Aguilera in this issue of BMC proceedings describes the process of applying for tenure at research-intensive R1/R2 institutions [2]. Foremost, a tenure at a PUI requires excellence in teaching, mentoring and a significant research component that engages undergraduates in meaningful ways leading to scholarly works and documented student success. PUI’s generally have higher teaching loads, advising loads, and service expectations combined with fewer financial resources available to fund undergraduate research programs than R1/R2 institutions [3, 4]. These hurdles present a unique challenge for faculty during the T&P process. The article by Dahlberg, King-Smith and Riggs in this issue gives important help on how to setup and build a productive research lab at a PUI [5].

Obtaining a PhD in the Biomedical or Biological Sciences requires one to learn how to conduct original research, communicate findings in the form of presentations or publications, and when mentored and supported appropriately, to apply to federal or private granting agencies. Success in these areas makes one a candidate for a career as a researcher in industry or in an academic setting. Indeed, many academic institutions require that an applicant demonstrate success in all these areas before taking the risk to invest in a new junior faculty because, even when successful, the indirect grant revenues generated by the faculty may not be enough to recover the start-up and research support spending by the university for many years [6]. However, excellence in research and grant writing does not guarantee that the individual will be successful through the faculty search and evaluation process that ultimately leads to the highly sought position of tenured faculty member or its equivalent. Despite efforts to diversify the academy, the hiring, tenure and promotion of women and racial/ethnic minorities still lag behind well-represented groups [7]. Although the percentage of women and faculty of color has gradually increased, the greatest growth in faculty has occurred among non-tenure track or part time faculty from 1993 to 2014 [7, 8]. It is important to recognize that implicit and explicit gender and racial bias still exist throughout the faculty evaluation process and that these biases result in increased stress and reduced success for T&P for women and women of color [9–12]. While we cannot offer specific remedies for these inequities, we aim to offer practical tips for navigating the T&P application process.

An essential question to address well before submitting a dossier for tenure and promotion (T&P) is to clearly understand and to articulate *what it means to be a tenured faculty at your institution*. Many new assistant professors have some familiarity with the expectations regarding scholarship, since graduate and postdoctoral training in the biological or biomedical sciences commonly occurs at research-intensive institutions [13]. However, their understanding and exposure to other areas of faculty development that include teaching, mentoring, advising, and service may be more limited [9]. Additionally, the need to document these activities continually and to engage in critical reflection throughout the process may not be apparent until it is too late [14]. A crucial component of successfully navigating the career milestone of obtaining tenure and promotion is the ability to identify and secure a network of mentors both internal and external to your institution that can guide and support you throughout the process [15–17].

Some of the key documents needed to apply for T&P may be found in the faculty handbook and is part of the formal communication process for new faculty hires. Generally, most T&P dossiers contain a cover letter, *curriculum vitae*, teaching statement, research statement, service statement, student teaching evaluations and evaluations from senior faculty committees and supervisors along with self-evaluation of teaching, scholarship and research. However, other important aspects of the T&P process can be institution specific, such as the impact of service, the importance of teaching relative to research activities or even the type of funding or publications that count towards T&P and, the specifics may be communicated through informal professional networks (IPN) [18, 19] Thus, the underrepresentation of women and minorities in STEM fields, especially at the higher faculty ranks, can represent a barrier to access these informal communication and support networks that also provide social support vital to job effectiveness and successful transition through the T&P process [18–20]. The lack of uniformity in definitions and requirements for tenure from institution to institution further complicates the matter [21, 22]. *A Practical Guide to Scientific Management for Postdocs and New Faculty* is a very useful resource to help recent hires as they begin their journey [23].

“Mentoring is a dynamic, reciprocal relationship between the mentor (or mentoring team) and mentee that promotes the mutual development and benefits for both” [24, 25]. Having access to experienced faculty mentors is critical throughout the T&P process since mentors can share unwritten norms and expectations and guide their junior colleagues on departmental politics and procedures. Mentors can also assist with some of the practical aspects of teaching, research and service that are part of the job. For example, they can observe your teaching or collaborate with the teaching of one your courses, they can share new research initiatives and opportunities or better yet, collaborate with you on a project or grant proposal. They may also assist you with navigating sensitive issues regarding students or colleagues. Importantly, they can advocate on your behalf and promote your work to others. Recognizing the critical roles of mentors in faculty success, many departments assign a formal “mentor” from within the department to help a pre-tenured faculty navigate the probationary period of their appointments, and it is a good practice for institutions that want to support Jr. faculty productivity and retention [24, 25]. Pfund et al. (2016) have split the multi-faceted dimensions of research mentoring into five domains: research, interpersonal, psychosocial and career, culturally responsive/diversity, and sponsorship [24]). Clearly, a junior faculty at a PUI will need mentoring in many other dimensions and it is unlikely that one person can serve as mentor for all. Thus, it is strongly recommended for mentees to seek and nurture relationships with several mentors [26].

Professional organizations such as the American Society for Cell Biology can also be a mechanism through which informal professional networks can lead to mentoring relationships [19]. Many professional societies have built in professional developing and networking sessions as part of the annual meeting programming. Formal mentoring programs such as the ADVANCE Scholar Program [26]), Faculty Research Education Development (FRED) Mentoring Program [27], Accomplishing Career Transitions Program (ACT) [28], and many more try to address some of the barriers encountered by underrepresented minority faculty and women by partnering Jr. faculty with successful external senior faculty to help develop the critical mentoring relationships. The National Research Mentoring Network (NRMN) is an important resource to connect you with a committed mentor to aid you through the T&P process and to assist you in develop effective mentoring and advocacy skills yourself [27, 29]. Part of documenting your growth as a faculty should include your growth as an effective mentee learning from your formal and informal mentors and developing your own mentoring practices that support mentee students and later, faculty (reviewed in [30]).

### Setting the stage

The journey to promotion and tenure begins on your first day as Assistant professor. Your employee orientation will direct you to a website that contains the Faculty/Employee Handbook. In addition to specifying the role and responsibility of faculty at the university, the Faculty/Employee Handbook describes the history and vision of the institution and identifies the university’s organizational structure and key university wide committees and councils. Make sure to read it. A clear understanding of the university organization should help you be a more effective university community member, which should ultimately facilitate your move up in rank and tenure success [23]. The university-level promotion and tenure guidelines and approval process may also be found there. Just as familiarity with the programmatic guidelines for a funding opportunity announcement (FOA) is indispensable for a successful grant application, understanding the faculty review process at the departmental, college, and university level is critical for future success. Don’t be afraid to ask trusted tenured faculty colleagues for tips regarding the T&P early in the process. Many are willing to share their dossiers to help support pre tenure colleagues if they are asked. Remember, you were hired to fulfill a critical need in the department and your success reflects well on the department’s ability to mentor, support and retain their junior colleagues.

At some institutions the tenure process is specified by the contract cycle, for example, a 1–1-1 (probationary period), followed by a 3 year contract. In this example, a person submits a dossier at the beginning of the 3rd year of the 3-year contract. However, self-evaluation of teaching effectiveness, mentoring research and scholarship are typically done on an annual basis. Use the annual self-evaluations as a formative experience, to reflect on your achievements and identify areas in teaching, scholarship or research that may need improvement. Even more importantly, use these self-evaluations to save and collect evidence of your accomplishments and progress: save these emails and files in a folder for future use in your T&P dossier. Indeed, we strongly recommend you develop this as a personal habit that you perform frequently throughout the year. In preparing your material for your annual evaluation during the probationary period (years 1–5) and for your T&P application (typically in year six), it is important to clearly articulate your role as a member of your university community and that you effectively communicate to your evaluators how your teaching, work and experiences enhance your department and the university community. Tenure (or its equivalent) results in a financial and legal obligation to you by the institution. Smaller institutions by necessity require significant collaboration among faculty and between faculty and students to support teaching and learning efforts and to produced research publications and presentations. Your dossier should demonstrate how your own professional goals and values align with that of your department and your institution. At times, the critical self-reflection and documentation needed to prepare your dossier can make you aware of conflicts and divergences between your career goals and those of your current institution, pushing you either to realign your goals, or to consider a future change of career or institution [31]. In any case, your thoroughness in putting together the documentation will either help you in the current promotion process or facilitate your career switch, if you choose to explore new opportunities. Your annual evaluations and ultimately your T&P portfolio must align with and demonstrate your growth in the three areas of faculty development: teaching, scholarship, and service.

### What goes into your package? The basic framework

Most T&P dossiers contain the following documentation: a cover letter, *curriculum vitae* (CV), teaching statement, research statement, and service statement that narrate your growth during your probationary period. Use the recommended (or required) template for the CV since it is the one that T&P committees are accustomed to reviewing. Updating the *vitae* format can be tedious so, update your CV to the university template sooner rather than later. Because teaching has a central role at PUIs, a selection of student teaching evaluations is required either in the dossier or are provided to the committee as part of the documentation process. Annual evaluations by key administrators such as department Chairs and/or Deans are also included. Thus, it is important to pay careful attention when documenting your annual progress and to keep careful records of how your activities fit into the evaluation framework. To make assembling the annual report simpler, keep a Word document with bullet points that you can easily update to have a running list of your activities and a separate list of research students. Ideally, the running list of activities would be organized in the same broad categories that are used to generate the annual activity reports. Letters of support from the chairperson or Dean and from the senior faculty evaluation committee in your department are also included. Additional letters of support from external evaluators may be solicited by you or by the Dean, the Chair, or the university’s T&P committee. Similar to submitting a list of potential reviewers for a research article, begin to assemble a list of potential outside reviewers early in the process and keep them abreast of your progress as a junior faculty. Start early, find senior faculty mentors or mentors that are internal and external to your university and obtain examples of a successful portfolio from someone who recently went through the process. If you have a joint appointment, ensure that you have examples of dossiers from each of your departments.

### Assembling the evidence

#### Teaching, advising and mentoring

Teaching excellence is not only a requirement for tenure and promotion at PUIs, it has a quintessential role in attracting and retaining students in STEM disciplines though their undergraduate degree and beyond [32]. Excellence in teaching is a key part of your development as a faculty member, and at PUIs the teaching, student mentoring/advising, and scholarship are intimately intertwined. Thus, it is very likely that some aspects of teaching will be relevant to all your narratives. A typical new assistant professor has much to learn about the essential role of faculty in teaching and learning. As a new STEM educator and mentor, learn to recognize students as collaborators in the learning process. Their diverse cultures and lived experiences strengthen our discipline and collaboration and close mentoring empowers them to acquire the confidence and necessary technical skills to persist in STEM [33]. Teaching includes pedagogy, course and curriculum development, student mentoring and advising, and the creation of innovative programs to assist teaching. The teaching statement in your T&P dossier should describe your teaching philosophy and journey as a teacher, and the evidence should clearly document your growth, efforts, and accomplishments in this area. Voice matters, be clear in your narratives, ultimately the T&P committees want to know what you teach, your approach to teaching it, and the outcome of your efforts [34, 35]. For example, if your teaching statement describes a particular workshop that was impactful to your teaching, then providing evidence of attending a workshop is less compelling than including syllabi or assessments that clearly incorporate a novel pedagogical practice in the classroom. Student evaluation excerpts or assessment data that corroborate the effectiveness of your particular approach adds an even richer dimension to the tapestry that you are creating. At a PUI, your approach to students doing research for credit can be a part of your teaching statement and the results of their efforts should be included in your scholarship statements especially if they present their work at local or national meetings. Awards earned by your students for their research or research presentations should be highlighted in your T&P portfolio. Weaving the story of your development as a teacher-scholar into your teaching statement makes for a better dossier that also serves to describe you more fully to the committee reading your portfolio. If your position includes formal academic advising, make sure to describe it in the portfolio and include any evaluations of your effectiveness relative to others in your department or college. Even if your position does not include a role as a formal academic adviser, student advising and mentoring can take many forms: mentoring student clubs, organizing student activities, assisting students in their career search or graduate school application, and mentoring your research students or thesis committee member. Sample letters of recommendation for students involved in your courses or in your research program can serve to illustrate your ability to partner with undergraduate students in meaningful research in your lab or course and can document your effectiveness as a letter writer to help students achieve their professional career goals. Student research awards at local or professional conferences are evidence of your teaching and research effectiveness.

While student evaluations of teaching have been recognized as imperfect tools to assess teaching quality [21, 36, 37] they remain the norm. Become familiar with the teaching evaluation form questions and check with more seasoned faculty to know which evaluation items carry more weight or are particularly important to your department and/or T&P committee. Your teaching section of the portfolio may be required or be expected to include teaching evaluations from several recent years. If permitted, consider summarizing teaching evaluation trends using a graph, table, or chart, and have your Chair sign off and certify the accuracy of the data. A simple figure summarizing you teaching evaluations for a particular course could show that you have steadily improved as an instructor or that you were great from the beginning and remember to report how your course evaluations compare relative to departmental averages. Do not hesitate to provide a narrative to your documentation. In some cases, it is appropriate to include selected student comments as evidence. Good feedback from diverse courses could be used to demonstrate your ability to engage with various student audiences. Even negative student comments could form a narrative of how you read and reflected on your students’ comments and incorporated suggestions or improvements into your courses but documented improvement over time is imperative. If there are issues, make sure to address them as well. For example, indicate if poor evaluations were due to mitigating circumstances such as taking on additional teaching/advising responsibilities due to the illness or death of a colleague in the department or the spread of a viral pandemic. Indicate some of the unique challenges or opportunities of the courses in your teaching repertoire.

Student teaching evaluations remain the most common method to evaluate teaching, even as questions about their usefulness as sole measure of teaching effectiveness have surfaced [36, 38, 39]. Studies have suggested they often suffer from low response rate and that they may reflect the students’ implicit bias and perceived course difficulty rather than teaching effectiveness [37]. Student teaching evaluations may also show gender and cultural biases that disproportionately affect women and minority faculty [40–42]. Still, for now, student teaching evaluations will continue to wield a big influence on your teaching assessment. Including peer-teaching evaluations by senior colleagues in your field or in Education and documenting student learning outcomes can provide additional support for your teaching effectiveness. Peer teaching evaluations are usually conducted during your probationary period; but they can be a great way to show that you are enhancing your skills as a teacher when you are going up for promotion. You might consider inviting a colleague to review your teaching if your institution does not do peer teaching evaluations.

#### Scholarship

The scope and amount of scholarship that are necessary for T&P varies among institutions. Departmental guidelines define the professional activities that constitute significant scholarly or creative contributions. If you hold a joint appointment, you will need to familiarize yourself with the guidelines for both departments. It is important to discuss openly the expectations regarding the number and types of scholarly publications and presentations that are needed for T&P with your Chair or Dean well in advance of your submission and to document your discussions. These discussions should also include a discussion about resources and release time that you will need to be successful in research. In some institutions, these discussions are codified into a Memorandum of Understanding (MOU) for tenure track faculty and are used to evaluate the faculty member’s progress. Again, seek out faculty mentors who can guide your through the process.

Like your teaching statement, your scholarship narratives should demonstrate your continued growth as a scholar and a recognized contributor to your field of study. It must also clearly describe the ways that you partner with and engage undergraduate students in meaningful research. High teaching loads and limited funds may make conducting research challenging; thus, creative solutions are sometimes needed. At PUIs, research projects with students may count for both teaching and scholarship. For example, evidence that documents developing course-based research projects can demonstrate a curricular innovation that both advances student learning and expands your research efforts [43, 44]. Meaningful hands-on research experiences combined with strong mentoring for undergrads promotes student learning and retention and is well known as a high-impact student experience [45, 46]. Publications with student co-authors are excellent evidence of mentoring and research productivity. Use professional networks like LinkedIn to follow your mentee’s accomplishments. Establishing and documenting collaborations with others in your field is a great way to increase your research productivity and make connections while enabling your students to gain experience and mentoring from other scientists [43, 47]. Bioinformatic programs that support student learning through research, such as the Genomics Education Partnership (GEP) [48] and Science Education Alliance-Phage Hunters Advancing Genomics and Evolutionary Science (SEA-PHAGES) (among others), help with networking and provide support for the integration of new advanced technology and resources into courses and research projects for faculty and students at PUIs [46, 48, 49]. Within your PUI, take advantage of every opportunity to get to know colleagues outside your discipline and consider collaborating with them on an inter- or multidisciplinary research or creative project. Do not overlook the opportunity to engage in research on STEM education itself – you are in the perfect institution to do so. Funded proposals, proposal submissions, travel awards, publications, presentations & talks are all evidence of your research accomplishments.

At many PUI’s, you may be a member of a department where you are the only specialist in your scientific sub-discipline. Thus, the ability to evaluate your research will partly rely on how well you communicate your research activities to colleagues outside of your specialty. Presenting an even greater communication challenge, the university wide committees that evaluate your promotion documents may include few to no scientists and none in your field. It is imperative to define your contributions in terms a layperson can understand – take the time to introduce the background of your scholarship, focus on the essential findings and their importance, and avoid the technical jargon that too easily creeps into our scientific writing. Consider adding simple figures to communicate key ideas and enlist the help of a more senior person well outside of your field to read your scholarship statement. Take their suggestions to heart. Clearly indicate whether your research or presentation went through a peer-review process and/or if it was solicited. While most scientific conference abstracts are not considered peer-reviewed, they still represent the product of significant scholarly work and communication [48]. Make sure to explain the authorship rules, as various disciplines have different rules concerning the order of authors in publications. Explain your contributions to publications with multiple authors and emphasize the hard work that earned you authorship.

Many smaller institutions lack resources to support a frequent seminar series with paid outside speakers, so do not hesitate to reach out to colleagues at other institutions (particularly other PUIs) and offer to give a seminar or a guest lecture. Also, reciprocate the gesture to invite outside speakers to enrich the research environment at your own institution. Don’t forget to acknowledge the achievements of your colleagues by nominating them for faculty awards and do not hesitate to nominate yourself for awards, if appropriate. Finally, share the challenges you experienced. Most science faculty had to adjust their research focus as they encountered various barriers to their original research approach at their PUIs. If it was an opportunity to expand one’s research interest into new fields or establish new collaborations, highlight it.

#### Institutional service

The service component of a promotion portfolio often comes with a question mark. How much is enough? What kind of service is deemed more relevant or significant by the T&P review committee? Many PUIs have service at their core origin and stated mission, and they highly tout its importance. However, when it comes to promotion, many T&P committees evaluate service as a box to be checked rather than an area for outstanding excellence [49, 50]. A growing movement is afoot to integrate aspects of service into scholarship [51], taking advantage of the door opened by Ernest Boyer’s suggestion to reimagine the definition of scholarship in *Scholarship Reconsidered* [52]. However, most universities still defer to the more traditional definitions of scholarship that fail to include service. Ambitious aspiring professors can fall into the trap of acquiescing to excessive and underappreciated service obligations that make it tougher for them to earn promotions. Stories of faculty taking on an outsized service burden, compared to their peers, not only find that they fail to be rewarded or accommodated for these efforts, but that their odds of being promoted are diminished as they have less time for the key twin pillars of teaching and scholarship [53, 54]. This is particularly true for women and underrepresented minority faculty that are sometimes asked to take on additional service roles to help their department or university to achieve their diversity and inclusion goals, and it has been termed a “minority tax” [55–58]. At its best, service allows you the opportunity to engage with the larger community, participate in university decision making, and provides opportunities for professional development through professional service. Service should advance the mission or goals of the University or larger scientific community and the service that you choose to do should also be meaningful to you without leading to burnout and stress [55]. Document the service activities that you engage in and discuss service loads with your colleagues and your supervisor to ensure that the amount of service is equitable compared to other faculty in your department during the annual review process [55].

Service opportunities exist at the department, college, university, and professional service to your discipline and to the community. Examples of service at the departmental level include serving on faculty search committees, curriculum committees, or any other established or ad hoc committee. University service may be through the Faculty Governance process (e.g. Faculty Senate) or other University committees. Service in university committees, faculty senate and otherwise, also helps you to meet and build critical relationships with faculty outside your department or college while learning more about the administrative side of your institution. Like the teaching and research statements, the service statement should tell a story or theme rather than detailing a list of disparate activities. For example, if you have a personal goal of increasing diversity and inclusion at your PUI, then highlight how the various service activities that you are engaged in helped to advance this aim. Participating in K-12 outreach activities, recruiting minority undergraduates, serving on search committees, organizing outside speakers, mentoring junior faculty or serving on diversity committees are all forms of faculty service that can also support a desire to advance diversity, equity and inclusion (DEI). The commitment to DEI can also be supported by evidence in the other sections of the dossier, for example, diversity related course content and inclusive pedagogy described in the teaching statement. Thus, engage in strategic service opportunities that align with your values and professional goals but also advances the vision of the university.

It is important to keep in mind that some service activities could end in publications or presentations that count toward your productivity as a scholar. If there are important lessons or significant findings from your service, take the time to write it up [59–61]. Including students in service learning is a high impact practice that incorporates experiential learning with community engagement into a course. These experiences can empower students, strengthen communities, and promote student learning and retention. Education-focused journals such as CBE-*Life Science Education* and *Journal of Molecular Biology Education* publish articles that describe and evaluate the effectiveness and outcomes of these activities on communities and students. Additionally, professional societies and funding agencies have specific funding opportunities for community engagement (NSF, ASCB) that allow you to connect some of your service activity to your scholarly or creative works.

#### Service outside of the PUI

Professional service to your discipline through your professional societies such as American Society of Cell Biology not only allows you to participate in service opportunities but also helps you to network and engage with scientists and peers outside of your immediate institution. Many of these societies offer multiple types of professional service opportunities. Some professional service opportunities require multi-year commitments, i.e. serving as a journal editor or science board member, while some are more limited in duration and scope, i.e. serving as a poster judge or abstract reviewer or mentor. Many of these service opportunities help you to develop leadership skills via formal and informal communication channels that will help you to move forward throughout your career [15, 62]. Professional service may also include serving as a peer reviewer for research articles or volunteering as a grant proposal reviewer. These professional service opportunities can help you to expand your professional network and help you learn about the grant and manuscript review process, improving your chances on your next grant applications or manuscript publications. Thus, while the experiences may be considered professional service, they are relevant to your research statement and your effort to develop as a scholar. Your service can also introduce your PUI to grant funders and program officers.

#### Potential pitfalls

One common error for junior faculty applying for T&P is that they do not know how the process works or when to apply. Another is not asking for help from trusted colleagues or mentors. Enlist trusted colleagues and your department chair to review your portfolio and heed their suggestions. Go outside your immediate sphere: take advantage of the contacts you made with faculty outside your discipline. For example, an Education School colleague could provide helpful feedback on your teaching statement, a fellow STEM faculty from another discipline could testify to your efforts to engage in initiatives that promote STEM education, and a colleague that served with you on a university-wide committee could share how you served as an invaluable colleague to advance university interests. If anyone who is evaluating your dossier is mentioned in it, run the section by her or him to ensure that your writing accurately reflects the collaboration. While some institutions allow outstanding faculty to apply early, you should carefully consider whether an early application works in your favor. Only apply early if you are permitted to according to the Faculty/Employee handbook, or if you are invited to apply early, and if your work and evaluations exceed the requirements for T&P. In addition, tenure delays due to family responsibilities or the Covid-19 pandemic etc., exist for a reason, so do not hesitate to use the opportunity if it helps you. Nonetheless, it is essential that you begin the work of preparing your dossier well before your intended submission date. Consider finding colleagues (which may be outside of your department) and start a writing group to support each other along the way. Starting early enables you to address any issues or gaps that may become apparent as you go through the process.

## Conclusions

### Final thoughts

In conclusion, while we sought to give a brief set of advice on how to better prepare for tenure and the first promotion to associate level at PUI institutions, our guide cannot possibly cover all the idiosyncrasies that may be found at your own institution. Our final and most essential advice is to look for mentorship as early as possible, since it takes several years of teaching, scholarship, and service to establish your competence and excellence in these areas.

## Abbreviations

PUI: Primarily Undergraduate Institutions
T&P: Tenure and Promotion
STEM: Science, Technology, Engineering, and Math
BMC: BioMed Central
R1: Doctoral Universities – Very high research activity
R2: Doctoral Universities – High research activity
IPN: Informal professional networks
ADVANCE: NSF funding mechanisms for Organizational Change for Gender Equity in STEM Academic Professions
FRED: Faculty Research Education Development
ACT: Accomplishing Career Transitions Program
NRMN: National Research Mentoring Network
FOA: Funding opportunity announcement
CV: *Curriculum vitae*
MOU: Memorandum of Understanding
SEA-PHAGES: Science Education Alliance-Phage Hunters Advancing Genomics and Evolutionary Science
GEP: Genomics Education Partnership
DEI: Diversity, Equity, and Inclusion
K-12: Kindergarten to 12th grade
NSF: National Science Foundation
ASCB: American Society for Cell Biology
